# Glioblastoma cells express crucial enzymes involved in androgen synthesis: 3β‐hydroxysteroid dehydrogenase, 17‐20α‐hydroxylase, 17β‐hydroxysteroid dehydrogenase and 5α‐reductase

**DOI:** 10.1002/edm2.289

**Published:** 2021-07-23

**Authors:** Jose Antonio Mondragón, Yesenia Serrano, Andrea Torres, Martin Orozco, Jose Segovia, Gabriel Manjarrez, Marta Catalina Romano

**Affiliations:** ^1^ Departamento de Fisiología Biofísica y Neurociencias Centro de Investigación y de Estudios Avanzados del IPN Ciudad de Mexico Mexico; ^2^ Unidad de Enfermedades Neurológicas Hospital de Especialidades México City México

**Keywords:** cancer, glioblastoma, neurosteroids, steroidogenic enzymes

## Abstract

Glioblastoma (GB) is the most common and aggressive primary brain tumour in adult humans. Therapeutic resistance and tumour recurrence after surgical removal contribute to poor prognosis for glioblastoma patients. Men are known to be more likely than women to develop an aggressive form of GB, and differences in sex steroids have emerged as a leading explanation for this finding. Studies indicate that the metabolism and proliferation of GB‐derived cells are increased by sex steroids, the expression of androgen receptors (ARs) and the synthesis of androgens and oestrogens, suggesting that these hormones have a role in the tumour pathogenesis. The expression of aromatase, the enzyme that converts androgens to oestrogens, has been reported in glial cells and GB cell lines. Thus, it was necessary to test whether the steroidogenic enzymes involved in androgen synthesis are expressed in GB cells. Therefore, here, we investigated the expression of four key enzymes involved in androgen synthesis in human‐derived GB cells. U87 cells were cultured in Dulbecco's modified Eagle medium plus foetal bovine serum and antibiotics on slides for immunocytochemistry or immunofluorescence. U87, LN229 and C6 cells were also cultured in multi‐well chambers to obtain proteins for Western blotting. We used primary antibodies against 3β‐hydroxysteroid dehydrogenase (3β‐HSD), 17α‐hydroxilase/17,20‐lyase (P450c17), 17β‐hydroxysteroid dehydrogenase (17β‐HSD) and 5α‐reductase. Immunocytochemistry, and immunofluorescence results revealed that glioblastoma cells express 3β‐HSD, P450c17, 17β‐HSD and 5α‐reductase proteins in their cytoplasm. Moreover, Western blot analyses revealed bands corresponding to the molecular weight of these four enzymes in the three GB cell lines. Thus, glioblastoma cells have the key enzymatic machinery necessary to synthesize androgens, and these enzymes might be useful targets for new therapeutic approaches.

## Graphical abstract

Glioblastoma (GB) is the most common and aggressive primary brain tumour in adult human, and androgens may play a role in its development.Therefore, here, we investigated the expression of four key enzymes involved in androgen synthesis in human‐derived GB cells.Immunocytochemistry and immunofluorescence results revealed that glioblastoma cells express 3β‐HSD, P450c17, 17β‐HSD and 5α‐reductase in their cytoplasm. Western blot analyses revealed bands corresponding to the molecular weight of these four enzymes.Thus, glioblastoma cells have the key enzymatic machinery necessary to synthesize androgens, and these enzymes might be useful targets for new therapeutic approaches.

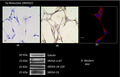

## 1 INTRODUCTION

Among local brain tumour types, glioma is the most aggressive. The World Health Organization (WHO) has proposed classifying gliomas into four grades based on a set of morphological criteria that determine the cell lineage.^1^ More recently, a molecular‐based classification (WHO, 2016) for GB was proposed, where isocitrate dehydrogenase wild‐type IDH that accounts for about 90% of cases, frequently corresponds with the clinically defined primary or *de novo* glioblastoma, and predominates in patients over 55 years old, and GB IDH‐mutant (accounting for 10% of cases), so‐called secondary glioblastoma with a history of prior lower grade diffuse glioma, occurs preferentially in younger patients.^2^ The treatments for those types of neoplasms consist mainly of surgery for tumour ablation, radiotherapy and chemotherapy, mainly with temozolomide (TMZ). Unfortunately, patient survival is poor because of frequent recurrences.

Several studies have proposed that steroid hormones play an important role in developing gliomas.^3^ Preliminary evidence was provided by statistics from the United States of America, where the incidence in the adult population is 50% higher in men than in women^4^, ^5^). Likewise, the expression of receptors to oestrogen, progesterone and androgens in glioma cell lines and tumour biopsies in GB cell lines has been reported (Gandhari et al., 2006,^6^, ^7^, ^40^,Chang et al. 2014,^7^

The synthesis of neurosteroids by the central nervous system (CNS) comprises several enzymes. Steroidogenesis in the CNS begins with cholesterol entry into the mitochondria, which is regulated by the protein StAR (steroidogenic acute regulatory protein), which captures cholesterol in the outer mitochondrial membrane and transfers it to the peripheral receptor to benzodiazepines (PBR) located in the mitochondrial membrane. This forms a channel that directs the cholesterol to the inner mitochondrial membrane, where the first steroidogenic enzyme is found, cholesterol desmolase, cytochrome P450scc (side‐chain cleavage), which synthesizes pregnenolone (P_5_) in the endoplasmic reticulum. Starting from cholesterol, the first step of the pathway is to synthesize pregnenolone (P5), which is then metabolized to various hormones. The 3β‐hydroxysteroid dehydrogenase delta 4–5 isomerase (3β‐HSD) is a key enzyme in these metabolic pathways and is necessary for producing the 3‐oxo‐group present in most steroid hormones. This enzyme is also expressed in SH‐SY5Y cells^8^ [9]. CYP17 A1 (also termed 17α‐hydroxylase/C17‐20‐lyase) catalyses the hydroxylation of C21 steroids to C19 steroids. CYP17 A1 is also required to form dehydroepiandrosterone (DHEA) and androstenedione, weak androgens that are converted to testosterone (T) and estrone (E_1_). The expression of CYP17A1 mRNAs in the nervous system is controversial, but has been reported in neurons and astrocytes of the rat brain and SH‐SY5Y cells.^8^, ^9^ The multifunctional enzyme 17β‐hydroxysteroid dehydrogenase catalyses the oxidation of neuroactive steroids in the brain, which are involved in brain development, neurogenesis, neural plasticity and neuroprotection and has an impact on brain diseases. The originally described functions of 17β‐hydroxysteroid dehydrogenases were to maintain the balance between the less potent (17‐Keto) and more potent (17β‐hydroxy) forms of oestrogen and androgens, also, they are involved in the metabolism of fatty acids and sterols (for a review, please see.^10^

The expression of the steroidogenic enzymes P450 cholesterol side‐chain‐cleavage and P450‐aromatase (P450arom) in glioma cell lines and tumour biopsies also accounts for the possible participation of steroid hormones in GBM. Papadoupulos et al.^11^ confirmed the presence of cytochrome P450 cholesterol side‐chain‐cleavage enzyme by immunoblotting in the mitochondria of the rat GB C6 cell line. The expression of the enzyme responsible for oestrogen biosynthesis, P450‐ aromatase (P450arom), in human and rat glioblastoma cell lines was described by Yague et al.^12^ Furthermore, Dueñas Jiménez et al.^13^ proposed this enzyme as a prognostic biomarker in astrocytoma patients.

The ability of GB‐derived cells to synthesize steroids has been reported by Papadopolous et al.^11^, who demonstrated that rat C6 cells transformed 22‐hydroxy cholesterol to pregnenolone, and by Melcangi et al.^14^, who reported that C6 and 1321 N1 human astrocytoma‐derived cells converted testosterone and progesterone into 5α‐reduced derivatives DHT and dihydroprogesterone (DHP). We have recently shown that U87 cells synthesize several androgens, and that the production can be inhibited by the 5α‐reductase inhibitors finasteride and dutasteride.^15^ Those results strongly suggest the presence of a panoply of enzymes involved in androgen synthesis.

The final products of steroidogenesis in each tissue depend on the enzymes that synthesize these hormones, such as P450‐cytochromes, steroid dehydrogenases and reductases.^16^ Baulieu^17^ described the CNS as a steroidogenic tissue and showed that steroids (eg pregnenolone (P_5_) and DHEA) and their sulphated forms (P_4_S and DHEAS) were found in higher concentrations in the brain than in the peripheral circulation of rodents Robel and Baulieu.^18^

The central nervous system (CNS) expresses most of the synthesizing enzymes or modifiers of steroids; most enzymes involved in the steroid synthesis have been documented in the rat brain and regions of the human brain.^19^, ^20^, ^21^, ^22^ The enzymatic pathways involved in neurosteroid synthesis and regulation have been extensively studied.^23^ Oligodendrocytes, astrocytes and neurons present steroidogenic capacity. However, it is important to note that these enzymes have distinct expression profiles and activities in neurons and glial cells.^9^ The conversion of androgens like testosterone to DHT, the potent AR agonist, results from 5α‐reductase activities. Therefore, these enzymes are essential for androgen synthesis in several classic steroidogenic tissues.^24^ Three types of this enzyme have been described, they are expressed in the normal prostate and are associated with prostate cancer initiation and progression^25^,the prostate 5α‐reductase mRNA levels of types 1 and 2 are regulated by testosterone and DHT.^25^

The 5α‐reductase isoforms 1 and 2 are express in the human brain where they participate in synthesis of neurosteroids.^22^ The 5α‐reductase type 1 is constitutively expressed in the rat brain,its expression is negatively regulated by T and DHT, while gene expression of 5α‐reductase type 2 is under the positive control of T and DHT.^26^

The physiological importance of 5α‐reductases in the brain derives from its ability to convert testosterone (T) to the potent androgen, dihydrotestosterone (DHT) and progesterone and deoxycorticosterone (DOC) to their respective 5α‐reduced derivatives, precursors of allopregnanolone and tetrahydro DOC, which are both strong allosteric modulators of the aminobutyric acid receptor (GABA_A_R).^27^ The implication of 5α‐reductases and aromatase in the growth of androgen and oestrogen‐sensitive tumours, such as prostate, breast and GB cancers, suggested that these steroidogenic enzymes are expressed in GB‐derived cells. Using RT‐qPCR, Zamora‐Sanchez et al.^28^ have shown 5α‐reductase mRNA expression in U87 cells.

We have recently shown that the androgens T_4_ and DHT, but not androstenedione, significantly increase GB metabolism, and that the effect of these androgens is inhibited by using the 5α‐reductase inhibitor dutasteride or the androgen receptor blocker finasteride.^29^ Since androgens have a role in GB development, it is important to have a deeper insight into the tumour steroidogenic ability, focusing on androgen synthesis. Here, we aimed to describe the expression of key steroidogenic enzymes involved in the androgen synthesis by GB‐derived cells.

## 2 MATERIALS AND METHODS

### 2.1 Cell culture

Human GB‐derived U87 cells were purchased from American Type Culture Collection (ATCC, Manassas, VA, USA). Cells (5–10^4^) were seeded in culture slides (Falcon, New York, USA), cultured in high‐glucose Dulbecco's modified Eagle medium (GIBCO BRL) supplemented with 10% foetal bovine serum (FBS, GIBCO BRL), 2 mM glutamine (Sigma) and 100 units‐µg penicillin‐streptomycin (GIBCO BRL) and maintained at 37°C in 95% air, 5% CO_2_. For the experiments, the complete medium was removed, and the cells were washed with phosphate‐buffered‐saline solution, pH 7.2 (PBS). For the Western blot analysis, the cells were seeded in multi‐well plates and cultured in the same conditions described above. LN 229 and C6 cells were cultured with the same methodology described for U87 cells.

### 2.2 Immunocytochemistry

The cultured cells were fixed in 4% paraformaldehyde and ethanol dehydrated. An antigenic unmask was carried using a 0.1 M citrate buffer solution, pH 6.0 (DECLERE, Cell Mark, Rocklin, CA, USA), followed by the inhibition of endoperoxidase activity with 5% H_2_O_2_. After washing the slides, they were incubated with specific primary antibodies for CYP17 (Rabbit Polyclonal CYP17A1 antibody, GeneTex), for 5α‐reductase Type2 **(**rabbit monoclonal [EPR6281(B)] to SRD5A2, Abcam) and antisera against 17β HSD type I (Raised in rabbit using purified type 1 from the human placenta) and 3β‐HSD (Raised in rabbit using purified type I human placental 3β‐HSD) at a dilution of 1:1000 in a commercial antibody diluent solution (Cell Marque) and incubated for 18 h at 4°C. Next, the sections were washed with PBS and incubated with the secondary antibody diluted in PBS and Triton X 100 (Biotinylated anti‐mouse‐rabbit IgG antibody) for 30 min. The cells were then incubated with avidin‐biotin complex for 15 min at room temperature and washed. Immunoreactivity was determined with a commercial kit (3,3‐diaminobenzidine and H_2_O_2_) according to Vector Laboratories’ protocol^30^ and mounted in ENTELLAN mounting media for microscopy. Photomicrographs were taken with a digital camera (Infinity 1‐Lumenera) with a 40× objective, using an Infinity 1‐Lumenera camera equipped with a 10× objective, aided by an Olympus microscope.

### 2.3 Immunofluorescence

For immunofluorescence observations, we fixed the slides as described for immunocytochemistry and exposed them to the same primary antibodies. After 24 h, the slides were washed with 0.1% PBS‐Tween and then incubated with Alexa Fluor 546 anti‐rabbit diluted to 1:100. The slides were mounted with ProLong Diamond Antifade Mountant for immunofluorescence and observed under a Leica TCS *SP8* Confocal Laser Scanning Microscope (Leica Microsystems) with a 40× objective.

### 2.4 Western Blotting

Cells were scrapped with a cell homogenizer and mixed for 30 s at 4°C in a solution of 50 mM Tris‐HCl, pH 7.4, plus protease inhibitors (Protease Inhibitor Cocktail, Sigma‐Aldrich Quimica SA de CV, Mexico). The samples were then centrifuged at 29,000 *g* for 15 min at 4°C. Protein concentration was determined by the bicinchoninic acid method. Then, 30 µg of protein were placed in each of the 1‐mm thick channels of an SDS‐polyacrylamide gel at 12%. The electrophoretic run was performed at 100 V for 2 h (Mini‐Protean Tetra System, BioRad). For protein electrotransference, the gels were mounted on nitrocellulose membranes, and the run was performed at 25 V for 45 min (Trans‐Blot SD Semi‐Dry Transfer Cell, BioRad). Nitrocellulose membranes with the transferred proteins were placed in a blocking solution (Millipore Chemiluminescent Blocker) at 50% for 30 min. The membranes were incubated with the same primary antibodies specific for 3β‐HSD, CYP17, 17β HSD and 5α reductase used for immunocytochemistry described above, at a dilution of 1:1000 in the same blocking solution. On the following day, membranes were incubated with a secondary anti‐rabbit antibody (BioRad) at a dilution of 1:5000 in blocking solution. Membranes were revealed with chemiluminescence (Millipore, corporation, Billerica, U.S.A) and exposed to a film (Hiperfilm). The internal control used was glyceraldehyde‐3‐phosphate dehydrogenase (GADPH) or tubulin. The bands obtained were analysed and quantified by densitometry using Image Studio Software (LI‐COR Biosciences).

## 3 RESULTS

**FIGURE 1:**
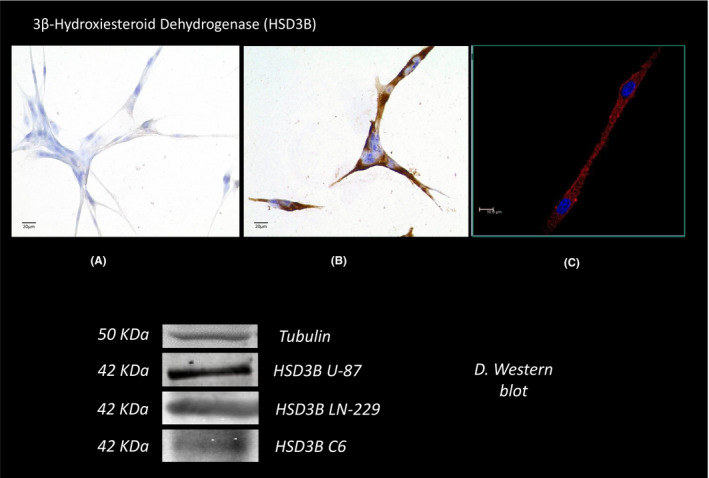
Micrographs illustrating immunostaining and protein expression for 3β‐hydroxysteroid dehydrogenase in GB‐derived U87 cells. Immunoreactivity by immunocytochemistry: (A) Negative control, (B) expression of the enzyme. (C) expression of the protein by immunofluorescence (400X). Western blot expression of the protein in U87, LN299 and C6 cells, and corresponding densitometric analysis. 400×

Figure 1 shows the abundant immunostaining for 3β‐HSD detected in the cytoplasm of human GB‐derived cells U87 using an immunocytochemistry technique (Figure 1B). The negative control is shown in Figure 1A. Confocal images obtained by immunofluorescent also detected the enzyme in the cytoplasm (Figure 1C), and 3D images revealed the lack of immunoreactivity in the nucleus (not shown). The expression of U87 protein (42 KDa MW) corresponding to 3β‐HSD can be observed in the Western blot analysis in Figure 1D. This figure also shows the expression of the 3β‐HSD protein in another GB cell lines, LN229 and C6.

**FIGURE 2:**
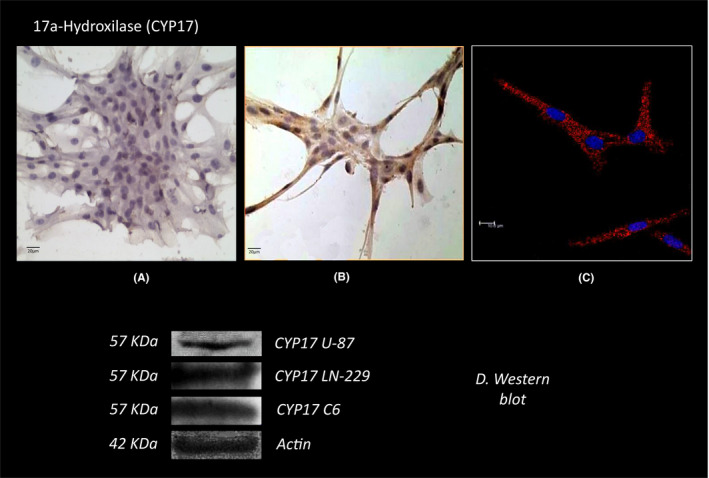
17α‐hydroxilase (CYP17) immunoreactivity in glioblastoma cells. (A) Negative control. (B) expression of the enzyme. (C) expression of the protein by immunofluorescence. Western blot expression of the protein by, and corresponding densitometry analysis. (400X). Western blot expression of the protein in U87, LN299 and C6 cells, and corresponding densitometric analysis

By immunocytochemistry, we detected the expression of 17α‐hydroxylase (CYP17), the enzyme that converts progesterone to 17OH‐progesterone and androstenedione in U87 cells (Figure 2B). Confocal images confirmed the cytoplasmic distribution of the enzyme in U87 cells (Figure 2C). Western blot analysis showed a band corresponding to the molecular weight of the of CYP17 protein (57 KDa MW, Figure 2D). This figure also shows the expression of the 3β‐HSD protein in another GB cell lines, LN229 and C6.

**FIGURE 3:**
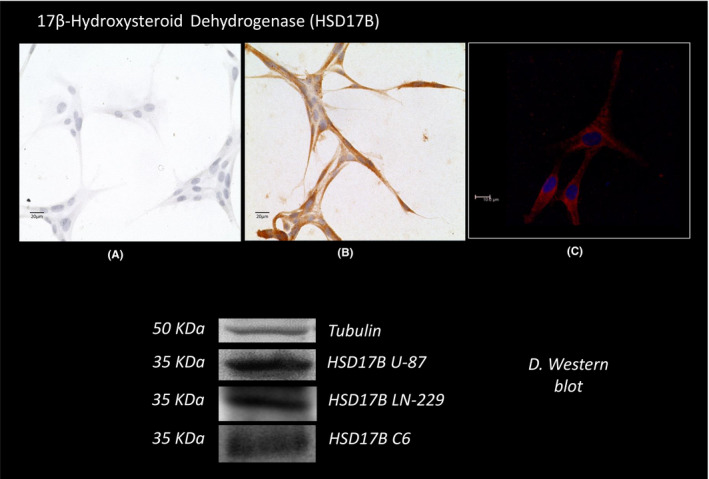
Light microscope micrograph illustrating 17β‐hydroxysteroid dehydrogenase expression as studied by immunocytochemistry, confocal microscopy, and Western blot. (A) Negative control. (B) expression of the enzyme. (C) expression of the protein by immunofluorescence. (400X). Western blot expression of the protein in U87, LN299 and C6 cells, and corresponding densitometric analysis

The 17β‐hydroxyesteroid dehydrogenase expression was found in the cytoplasm of U87 cells exposed to a specific antibody by immunocytochemistry (Figure 3B). Figure 3C displays the immunofluorescent images obtained by confocal microscopy. Both techniques showed a cytoplasmic distribution of the enzyme. Western blotting showed a band corresponding to the molecular weight of the protein (35 KDa MW, Figure 3D). This figure also shows that the expression of the 3β‐HSD protein is also present in another GB cell lines, LN229 and C6.

**FIGURE 4:**
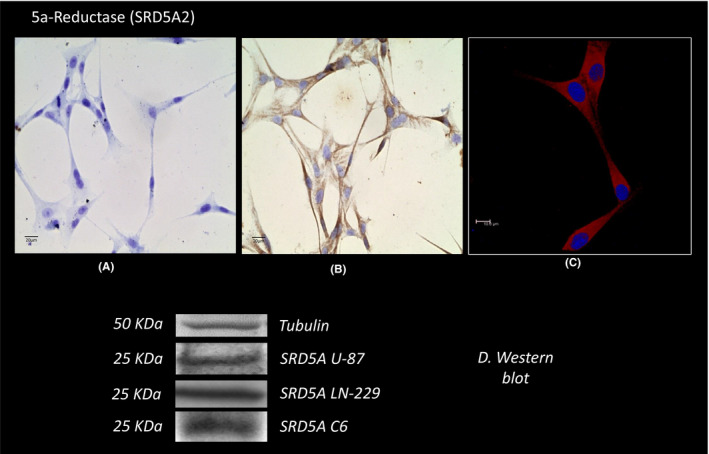
Micrographs illustrating immunostaining and protein expression for 5α‐reductase Type 1 in GB‐derived U87 cells. Immunoreactivity by immunocytochemistry: (A) Negative control, (B) expression of the enzyme. (C) expression of the protein by immunofluorescence. Western blot expression of the protein in U87, LN299 and C6 cells, and corresponding densitometric analysis

The expression of 5α‐reductase type 2, an enzyme that converts T to the potent androgen DHT, is present in the cytoplasm of U 87 cells, as shown by immunocytochemistry (Figure 4B). The enzyme was also observed in confocal microscopy images (Figure 4C). Western blot analysis detected the expression of the 5α‐reductase protein (25 KDa MW) that can be observed in Figure 4D. This figure also shows that L229 and C6 cells express the protein.

**FIGURE 5:**
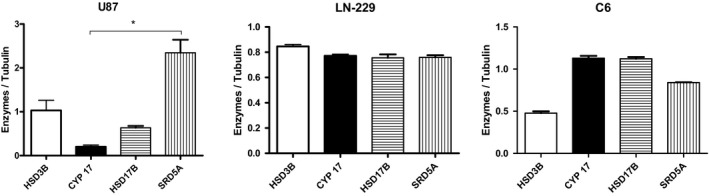
Densitometry corresponding to the expression of 3β‐hydroxysteroid dehydrogenase, 17α‐hydroxilase (CYP17), 17β‐hydroxysteroid dehydrogenase and 5α‐reductase in U87, LN229 and C6 GB‐derived cells. Data are presented as means ± SD. *N* = 3. ANOVA one way was followed by Kruskal‐Wallis and Dunn´s Multiple comparison tests. GraphPad Prism 5.01. *p < .05

Figure 5 shows the densitometric analysis of the expression of the four steroidogenic enzymes included in our study in U87, LN229 and C6 cells lines. The two human and the rat GB cell lines expressed the four enzymes, although their expression level was different, the prevalence of the investigated 5α‐reductase in U87 cells was evidenced by the statistical analysis.

## 4 DISCUSSION

As described in the Introduction, several authors have shown that normal glial cells synthesize androgens and oestrogens and express steroidogenic enzymes (P450c17, 3β‐HSD, 17β‐HSD and P450 aromatase).^9^ These findings suggest that the expression of these enzymes can be found in transformed glial cells. Supporting this possibility, our own studies and those of other authors have found that GB‐derived cells synthesize androgens and oestrogens,for the synthesis of these sex steroids, the action of several steroidogenic enzymes is essential, including 3β‐HSD, 17‐20α‐lyase, 17β‐hydroxysteroid dehydrogenase and 5α‐reductase. The inhibition of androgen synthesis caused by inhibitory drugs such as finasteride and dutasteride, strongly suggested the presence of specific enzymes in U87 GB‐derived cells.^15^ Following these findings, we have investigated the expression and distribution of the key enzymes directly involved in androgen synthesis in cultured GB‐derived cells using different techniques and confirmed their presence in two other cell lines by Western blot.

The expression of 3β‐HSD found in U87 cells has been reported in cultured normal glial cells^31^, and the results of our immunocytochemical techniques show that this enzymes are preserved in GB‐derived cells. This finding supports the reported steroidogenic capacity of C6 and U87 cells to provide steroids by transforming pregnenolone, DHEA and androstenediol to its metabolites.^15^ Our Western blotting analyses further demonstrated the presence of the enzyme protein in U87, LN229 and C6 GB‐derived cells. Two isoforms of 3β‐HSD have been characterized in humans,type 1 is mainly expressed in the placenta, and type 2 in the adrenal cortex and gonads.^32^, ^33^, ^34^ Immunostaining using an antibody against 3β‐HSD type 1 shows that U87, LN229 and C6 cells express this isoform in the cytoplasm.

In the brain, DHEA and androstenedione are synthesized from pregnenolone and progesterone, respectively. The expression of the immunoreactive protein P450‐Cyp 17 has been found in neurons of the hippocampus, where it localizes in pyramidal neurons, granule cells of the dentate gyrus (Hojo et al., 2004), hypothalamic periventricular neurons and Purkinje cells.^35^ Furthermore, the immunocytochemical expression of P450‐Cyp17 in astrocytes was found in the rat brain by Zwain and Yen.^9^ We have previously reported that U87 cells synthesize DHEA and androstenedione,^15^ a finding that suggested the presence of P450‐Cyp 17 in these cells. The subcellular expression of this enzyme is now confirmed by immunocytochemical techniques and by the expression of the protein by Western blot analysis.

The activity of 17beta‐HSD has been suspected based on brain tissue capacity to synthesize androgens and oestrogens^18^, ^23^. We have previously found the transformation of A_4_ to testosterone, and DHEA to androstenediol by U87‐derived GB cells^15^,therefore, the enzyme should be necessarily present in these cells, as we show in this study using immunohistochemical procedures. The expression of the enzyme found by Western blot further confirms the presence of the protein in GB cells.

Type 1 17beta‐HSD also participates in the synthesis of oestrogens, transforming estrone to estradiol and vice‐versa.^36^ Our study showed that the type 1 17beta‐HSD antibody recognized the GB enzyme in the immunocytochemical and Western blot studies presented here, confirming that type 1 is present in the cells. This type facilitates the reduction in DHEA to androstenediol, and DHT to 3α and 3β‐diol, and reactions that other authors and we have shown to occur in GB cells.^37^

We and others have previously shown that the potent androgens testosterone and DHT increased the metabolism and proliferation of GB‐derived cells^29^, ^38^, and these steroids positively control the gene expression of 5α‐reductase type 2.^26^ The 5α‐reductase protein was highly expressed in glioblastoma‐derived cells if compared with the expression of the three other enzymes investigated in this study, and a finding that could be explained by the positive control of the enzyme by the abundant androgens synthesized by these cells.

As we have shown studying the effect of androgens on U87 cells by MTT, testosterone and the strong androgen DHT, but not androstenedione, significantly enhance the metabolism, proliferation and invasion of U87 GB‐derived cells.^29^ Rodriguez‐Lozano et al.^39^ have shown that testosterone, promotes cell proliferation, migration and invasion of GB‐derived cells through the androgen receptors. Therefore, the expression of 5α‐reductase would be essential to provide the cells with potent androgens necessary for cell metabolism. We recently showed that dutasteride, or the combination of this 5α‐reductase inhibitor with the AR blockers, cyproterone and flutamide, significantly reduced the metabolism, cell proliferation and migration capacity of U87,^29^ findings that strongly support the presence of 5α‐reductases found in these cells.

The results presented here indicate that U87 cells maintain the steroidogenic enzymes present in glial cells. Therefore, U87 cells have the machinery necessary to synthesize androgens and other neurosteroids that might have a role in the GB development. These findings were also shown in LN229 and C6 cells. These enzymes might be considered as therapeutic targets for the treatment of human glioblastomas.

## CONFLICT OF INTEREST

We declare that there is notconflict of interest

## AUTHOR CONTRIBUTIONS

Jose Antonio Mondragón: formalAnalysis (equal); investigation (equal); methodology (equal). Yesenia C Serrano: investigation (equal); methodology (equal). Andrea C Torres: methodology (equal). Martin C Orozco: investigation (equal); methodology (equal). Jose C Segovia: conceptualization (equal); supervision (equal). Gabriel Manjarrez: conceptualization (equal); investigation (equal); methodology (equal). Marta Catalina Romano Pardo: conceptualization (lead); dataCuration (lead); formalAnalysis (equal); fundingAcquisition (lead); investigation (equal); writingOriginalDraft (lead); writingReviewEditing (lead).

## Data Availability

The data that support the findings of this study are openly available in [repository name e.g “figshare”] at https://www.doi.org/ [doi], reference number [reference number].
